# Comparative Effects of Oral Pregabalin, Intravenous Magnesium Sulphate, and Their Combination Given Preoperatively on Acute Post-thoracotomy Pain: A Double-Blinded Randomised Study

**DOI:** 10.7759/cureus.93810

**Published:** 2025-10-04

**Authors:** Kriti Bhandari, Bharat Choudhary, Shikha Soni, Garima Karamchandani, Pranay Bhaiya, Rakesh Karnawat, Subhash Balara

**Affiliations:** 1 Department of Anaesthesiology, Dr. Sampurnanand Medical College, Jodhpur, IND; 2 Department of Paediatrics, Dr. Sampurnanand Medical College, Jodhpur, IND; 3 Department of Cardiothoracic Surgery, Dr. Sampurnanand Medical College, Jodhpur, IND

**Keywords:** analgesic adjuvants, magnesium sulphate, opioid-sparing effect, post-thoracotomy discomfort, pregabalin

## Abstract

Introduction: Post-thoracotomy pain can lead to psychological distress and impair pulmonary function. Effective perioperative pain management is essential to prevent long-term complications. This study assesses the analgesic benefits of intravenous magnesium sulphate and preoperative oral pregabalin, both separately and in combination, in the treatment of acute post-thoracotomy pain.

Methods: Following ethical permission, 75 patients undergoing thoracotomy who were between the ages of 19 and 70 and had Physical Status II or III according to the American Society of Anesthesiologists were assigned to three groups at random: Group MP received 100 ml of normal saline supplemented with 50 mg/kg of intravenous magnesium sulphate and 300 mg of oral pregabalin, Group M received a placebo capsule and 50 mg/kg intravenous magnesium sulphate, and Group P received intravenous saline and 300 mg oral pregabalin. Intravenous infusions were given half an hour before surgery, and oral medicines were given an hour before.

Primary objective: This study aimed to compare 24-hour total morphine consumption (basal patient-controlled analgesia (PCA) infusion + patient-activated boluses) among pregabalin, magnesium sulphate, and combination groups.

Secondary objectives: This study aimed to compare postoperative Visual Analogue Scale (VAS) pain scores (one, two, four, six, eight, 12, 24 hours), duration of analgesia (time to first rescue), number of rescue boluses, incidence of postoperative nausea and vomiting (PONV) requiring treatment, Ramsay Sedation Scale scores, and patient satisfaction.

Results: In the first 24 hours after surgery, Group MP consumed the least amount of morphine (25.44 ± 2.50 mg), followed by Group P (27.00 ± 2.85 mg) and Group M (28.48 ± 2.90 mg). Group MP also had the lowest VAS scores, frequency of rescue analgesia, and incidence of PONV, with Group M showing the highest values (p < 0.001)*.*

Conclusion: Pregabalin and magnesium sulphate administered together prior to surgery successfully lower postoperative pain and the need for opioids in thoracotomy patients.

## Introduction

Thoracotomy is a surgical procedure involving access to intrathoracic organs such as the heart, lungs, esophagus, thorax, and aorta. This procedure entails cutting through multiple muscle layers, resecting or fracturing ribs, dislocating costovertebral joints, injuring intercostal nerves, and irritating the pleura through chest tubes and continuous respiratory motion [1,2]. Significant psychological suffering and pulmonary problems result from inadequate pain management following thoracotomy. When deep breathing is inhibited by pain, the expiratory muscles reflexively contract, reducing functional residual capacity and causing atelectasis, both of which contribute to hypoxaemia and ventilation-perfusion mismatch [1,2]. Furthermore, the physiological stress caused by pain triggers the sympathetic and neuroendocrine systems, which compromise immune and coagulation responses and raise morbidity and mortality [1,3].

Effective post-thoracotomy pain control is therefore essential for both patient comfort and respiratory function, allowing patients to breathe deeply, prevent complications, and begin mobilisation sooner [1-3]. Thoracic epidural analgesia is considered the gold standard for reducing post-thoracotomy pain, but its effectiveness is often limited. It frequently fails to relieve referred shoulder pain caused by the irritation of the C3-C5 nerve roots [1,4]. In addition, thoracic epidurals carry risks such as dural puncture, infection, spinal cord injury from haematoma or abscess, block failure, and systemic toxicity from local anaesthetics. These hazards are further increased in patients receiving antithrombotic therapy [4].

Despite their widespread use, systemic opioids also have drawbacks, including paradoxical hyperalgesia, respiratory depression, and postoperative nausea and vomiting (PONV), which can delay postoperative recovery and discharge [2-4]. For these reasons, opioid-based regimens frequently fall short of providing sufficient postoperative pain control.

Pre-emptive and multimodal analgesic strategies, administering complementary non-opioid agents before surgical nociception, have been shown to blunt central sensitisation and reduce perioperative opioid requirements [2,4,5]. Among such agents, pregabalin reduces excitatory neurotransmitter release via α2δ calcium-channel modulation, while magnesium antagonises N-methyl-D-aspartate (NMDA) receptors and attenuates central sensitisation [5-7]. Both drugs have demonstrated perioperative analgesic benefits when given before surgery in various settings [5-7].

Objective

This study aimed to evaluate the analgesic effectiveness of intravenous magnesium sulphate, oral pregabalin given before surgery, and their combination in treating acute post-thoracotomy pain.

## Materials and methods

Study design and ethical approval

This double-blind, randomised trial was conducted in the cardiothoracic operating room of Mathura Das Mathur Hospital at the Anaesthesiology Department of Dr. Sampurnanand Medical College in Jodhpur, India, following registration from the Clinical Trials Registry-India (CTRI) (registration number: CTRI/2023/08/057084) and approval from the Institutional Ethics Committee of Dr. Sampurnanand Medical College (approval number: SNMC/AcadCT/IEC/2023/Plan/714). Prior to enrolment, all subjects provided written informed consent.

Participants and randomisation

Using a computer-generated randomisation table, 75 patients of either sex, aged 19-70 years, who were haemodynamically stable, designated as Physical Status II or III by the American Society of Anesthesiologists (ASA PS), and cleared by pre-anaesthetic evaluation, were randomised equally into three groups of 25. The sample size was calculated based on the methodology reported by Abdelgalil et al., who used a similar randomised design with a 90% power and a type I error (α) of 0.05 [7]. Using opaque, sealed envelopes opened immediately before drug administration, group allocation concealment was preserved. Both patients and postoperative data collectors were blinded to group assignment.

Exclusion criteria

Exclusion criteria included known hypersensitivity or contraindication to study drugs, chronic use of pregabalin, gabapentin, or opioids, psychiatric disorders (including patients on antidepressant or antipsychotic medications), active addiction to alcohol or drugs, pre-existing pain at rest defined as a baseline Visual Analogue Scale (VAS) score >0 during preoperative assessment, hepatic or renal dysfunction, and surgeries lasting longer than four hours.

Outcomes

The primary outcome was total morphine consumption in the first 24 hours after surgery, defined as the sum of basal patient-controlled analgesia (PCA) infusion plus all patient-activated bolus doses. Secondary outcomes were as follows: (1) postoperative pain scores on the VAS at prespecified intervals (one, two, four, six, eight, 12, 24 hours); (2) duration of analgesia (time to first rescue bolus); (3) number of rescue boluses in 24 hours; (4) incidence of PONV requiring treatment; (5) Ramsay Sedation Scale (RSS) score at the same intervals; and (6) patient satisfaction measured by a 4-point Likert scale.

Preoperative preparation

Baseline oxygen saturation, heart rate, and blood pressure were recorded. The VAS and use of the PCA pump were explained to the patients. Nursing staff prepared the magnesium sulphate infusion by dissolving a 50% magnesium sulphate solution (500 mg/ml) in 100 ml of normal saline, infused over 10 minutes starting 30 minutes before induction to ensure adequate plasma concentrations at incision, following the approach described by Kiran et al. [8]. The Clinical Pharmacy Department created sterile placebo capsules by substituting sugar for the pregabalin capsule contents. A dose of 300 mg pregabalin was administered orally one hour prior to induction to allow sufficient absorption and peak effect during surgery, based on Bornemann-Cimenti et al. [9].

Interventions

Group MP received 300 mg oral pregabalin and intravenous magnesium sulphate 50 mg/kg diluted in 100 ml saline. Group P received 300 mg oral pregabalin plus an intravenous placebo (100 ml saline). Group M received a placebo capsule plus 50 mg/kg intravenous magnesium sulphate in 100 ml saline. Oral medication was taken one hour before surgery; infusions were given over 10 minutes 30 minutes before induction.

Anaesthesia management

All groups received identical anaesthesia. Standard ASA monitoring was started upon arrival in the operating room. Radial artery catheterisation was performed under local anaesthesia, and Ringer's lactate infusion was initiated. Premedication included fentanyl (2 µg/kg), midazolam (1 mg), and lignocaine 2% (1.5 mg/kg). Anaesthesia was induced with propofol (2 mg/kg) and rocuronium (1.2 mg/kg) after three minutes of preoxygenation with 100% oxygen. Double-lumen tubes were inserted after 1.5 minutes of mask ventilation. Anaesthesia was maintained with intermittent rocuronium (0.1 mg/kg), a continuous fentanyl infusion (1 µg/kg/hr), and inspiratory sevoflurane (1.5-2%), corresponding to about 1.0 MAC for patients in this age group. After surgery, patients were extubated and transferred to the cardiac ICU once the neuromuscular blockade was reversed with intravenous sugammadex (2 mg/kg).

Postoperative analgesia protocol

Immediately after extubation in the ICU, each patient was connected to a morphine PCA device containing 10 mg morphine sulphate + 8 mg ondansetron diluted to 50 ml (0.2 mg/ml). Basal infusion was 5 ml/hr (1 mg/hr; total basal dose = 24 mg/24 hr). Patients could self-administer 5 ml boluses (1 mg morphine) with a 15-minute lockout. Total morphine consumption was calculated as basal plus bolus doses.

Data collection

Total morphine consumption, rescue analgesia frequency, VAS scores, PONV incidence, RSS score [10], and baseline haemodynamics were recorded over 24 hours. Outcome measures were selected following Abdelgalil et al. [7]. Measurements were hourly for the first six hours, every two hours for the next six, and every four hours for the remaining 12.

Statistical analysis

Data were analysed using MedCalc (version 22.00) (MedCalc Software Ltd, Ostend, Belgium). Continuous variables are presented as mean ± SD and categorical data as number (percentage). Group comparisons used one-way analysis of variance (ANOVA), unpaired t-tests, Mann-Whitney U test, or Kruskal-Wallis tests as appropriate. Significance was set at p < 0.05.

## Results

Participant flow and baseline characteristics

**Figure 1:**
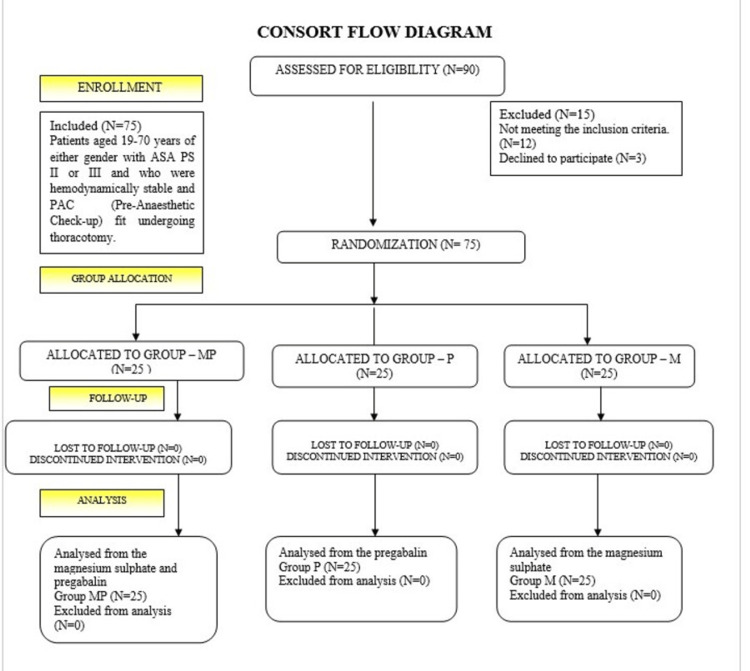
CONSORT flowchart of the enrolled patients throughout the study. CONSORT: Consolidated Standards of Reporting Trials; ASA PS: American Society of Anesthesiologists Physical Status

Between March 2023 and March 2024, 90 patients were recruited for thoracotomy surgery. Seventy-five of these patients fulfilled the requirements for inclusion and finished the research. Figure 1 displays the Consolidated Standards of Reporting Trials (CONSORT) flowchart that describes patient enrolment and allocation. There were no differences between the baseline demographics and haemodynamic variables of the three groups (including gender, age, height, weight, and ASA Physical Status) and surgical length (Table 1).

**Table 1:** Baseline demographic and clinical characteristics. Data are presented as mean ± SD for continuous variables and number (percentage) for categorical variables. Continuous variables were compared using one-way ANOVA or unpaired t-test, as appropriate, and categorical variables were analysed using the chi-squared test. The relevant test statistic and p-value for each variable are indicated in the table. A p-value of <0.05 was considered statistically significant. n: number of patients in the group; SD: standard deviation; M: male; F: female; ANOVA: analysis of variance; ASA: American Society of Anesthesiologists

Variable	Group M (mean ± SD/n, %)	Group P (mean ± SD/n, %)	Group MP (mean ± SD/n, %)	Total (n=75)	Statistical test (test statistic, p-value)
Number of patients	25	25	25	75	-
Age (years)	57.72 ± 11.48	57.24 ± 11.68	57.80 ± 12.47	57.59 ± 11.77	ANOVA (F, 0.87)
Gender (M/F)	17 (68%)/8 (32%)	16 (64%)/9 (36%)	17 (68%)/8 (32%)	50 (67%)/25 (33%)	Chi-square (0.120, 0.941)
Weight (kg)	67.48 ± 9.39	68.20 ± 12.10	66.12 ± 8.60	67.27 ± 10.08	Unpaired t-test (t, 0.82)
Height (cm)	166.48 ± 8.22	168.20 ± 8.85	165.56 ± 8.55	166.75 ± 8.55	Unpaired t-test (t, 0.48)
ASA Physical Status II/III	14 (56%)/11 (44%)	15 (60%)/10 (40%)	17 (68%)/8 (32%)	46 (61%)/29 (39%)	Chi-square (0.787, 0.674)
Duration of surgery (min)	228.48 ± 48.71	240.80 ± 71.73	232.40 ± 57.86	233.89 ± 59.26	ANOVA (F, 0.65)

Primary outcomes

**Figure 2:**
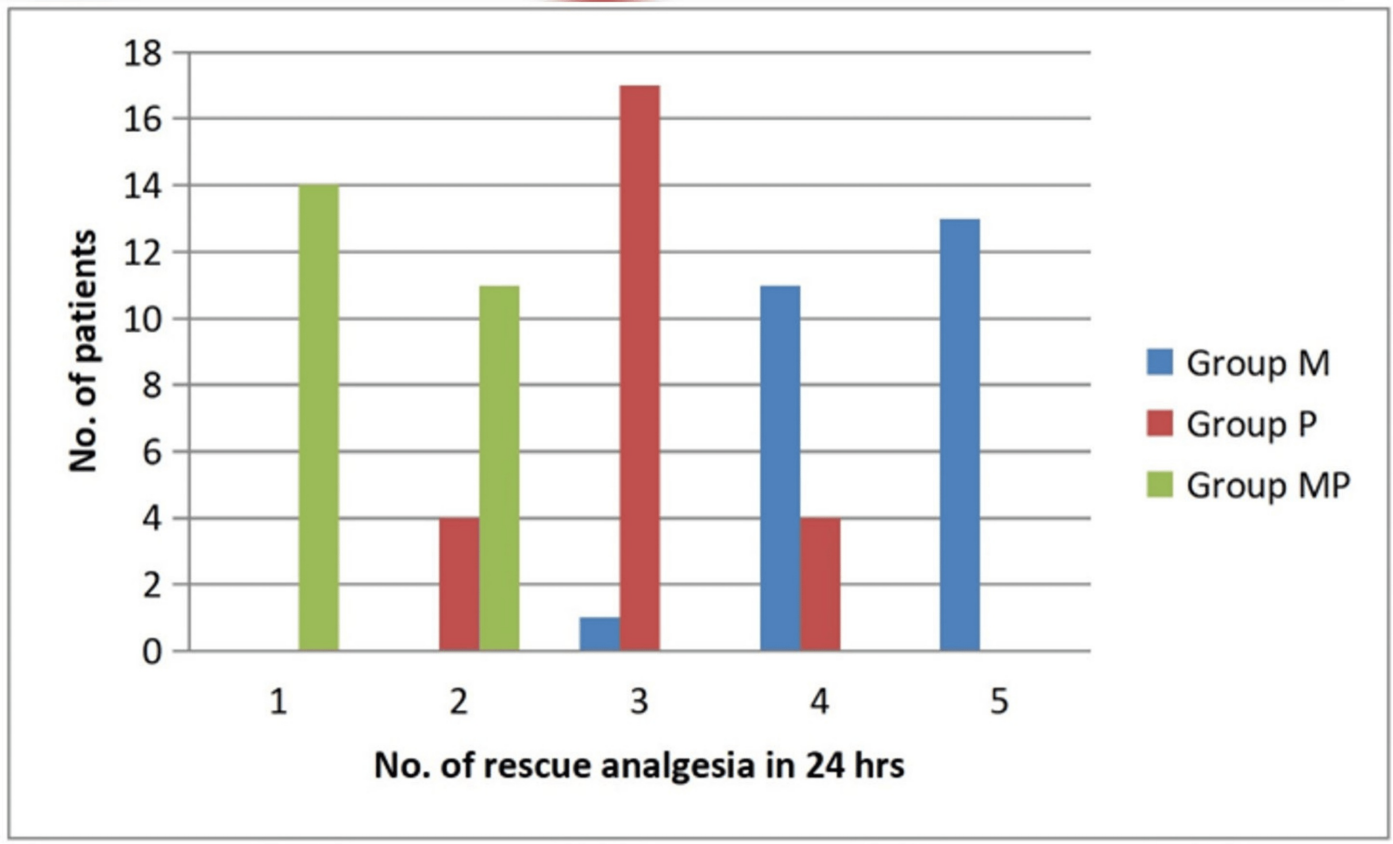
Bar chart comparing total morphine consumption (mg) in 24 hours among the three treatment groups, showing significantly lower consumption in Group MP.

Compared to patients receiving pregabalin alone (Group P) or magnesium sulphate alone (Group M), patients undergoing the combination therapy of oral pregabalin and intravenous magnesium sulphate (Group MP) consumed substantially less morphine during the first 24 postoperative hours (Figure 2). The mean 24-hour total morphine consumption (mean ± SD; 95% CI) was as follows: Group M, 28.48 ± 2.90 mg (27.28-29.68); Group P, 27.00 ± 2.85 mg (25.82-28.18); and Group MP, 25.44 ± 2.50 mg (24.41-26.47). One-way ANOVA confirmed a significant difference among groups: F(2,72) = 7.608 and p = 0.001. Post hoc tests showed the following: M vs MP (p = 0.0001); P vs MP (p = 0.045); and M vs P (p = 0.075). For transparency, basal, bolus, and total morphine consumption values are detailed in Table 2.

**Table 2:** Basal, bolus, and total morphine consumption in 24 hours. Values are mean ± SD unless otherwise indicated. Basal infusion was 1 mg/hour × 24 hours = 24 mg for all groups. Total = basal + bolus. 95% CI calculated for total morphine. SD: standard deviation

Group	Basal morphine (mg, fixed)	Bolus morphine (mg, mean ± SD)	Total morphine (mg, mean ± SD; 95% CI)
Group M	24	4.48 ± 2.90	28.48 ± 2.90 (27.28-29.68)
Group P	24	3.00 ± 2.85	27.00 ± 2.85 (25.82-28.18)
Group MP	24	1.44 ± 2.50	25.44 ± 2.50 (24.41-26.47)

Secondary outcomes

**Figure 3:**
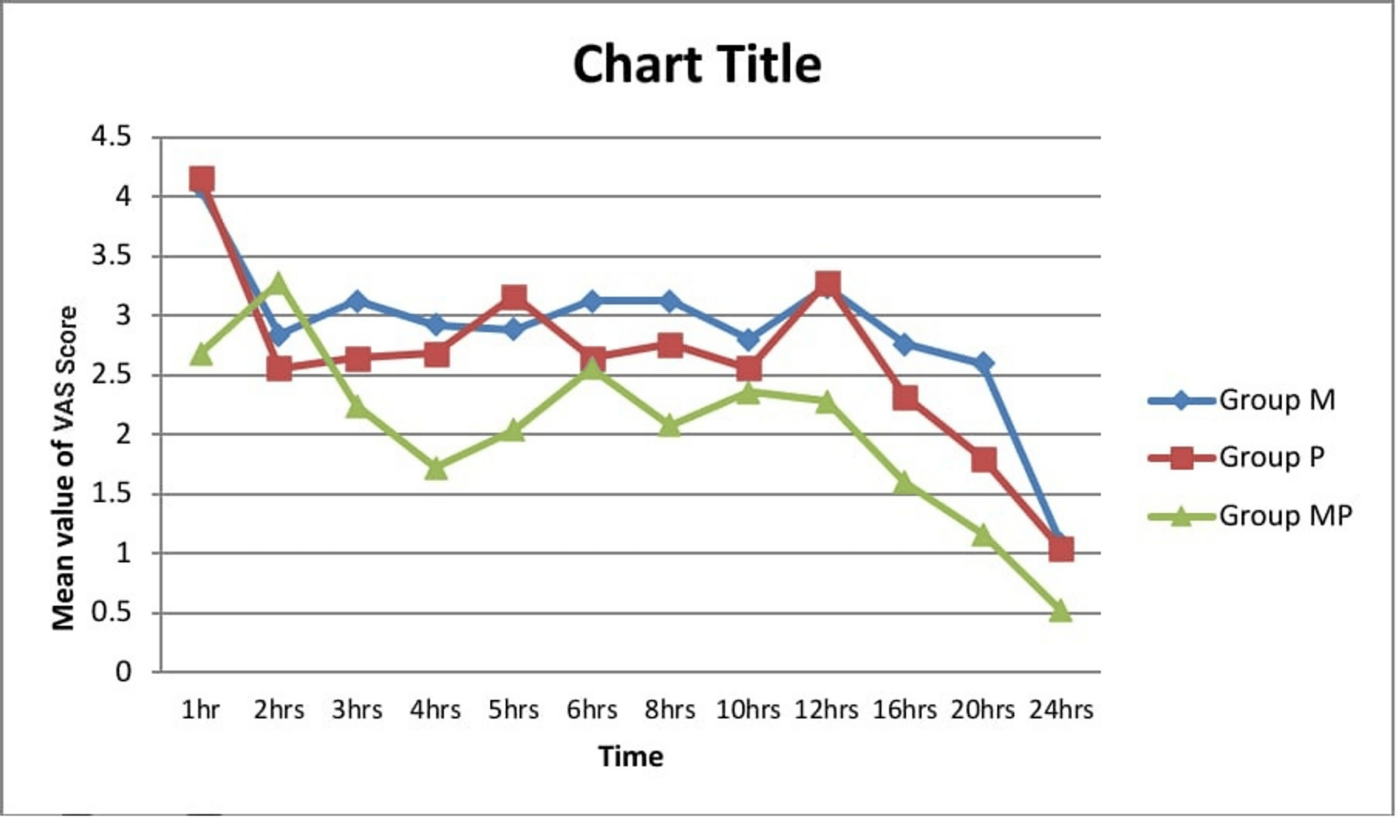
Line graph illustrating postoperative VAS pain scores over 24 hours in the three groups, with consistently lower scores in Group MP. VAS: Visual Analogue Scale

The VAS, which measures postoperative pain, showed that the median pain levels in all groups stayed at or below 4 for the full 24‑hour period. As demonstrated by a mean VAS of 2.68 ± 0.56 one hour after surgery, Group MP consistently reported the lowest pain levels, while Group P and Group M reported mean VAS scores of 4.16 ± 0.75 and 4.08 ± 0.91, respectively (Table 3 and Figure 3). Overall pain levels showed the following trend: Group M > Group P > Group MP. There were no discernible variations in the haemodynamic parameters following surgery.

**Table 3:** VAS scores postoperatively Data are presented as mean ± SD. Comparison of VAS scores between groups was performed using the Mann-Whitney U test. A p-value of <0.05 was considered statistically significant. VAS: Visual Analogue Scale; SD: standard deviation

Group	Mean VAS ± SD
Group M	4.08 ± 0.91
Group P	4.16 ± 0.75
Group MP	2.68 ± 0.56

Adverse events

**Figure 4:**
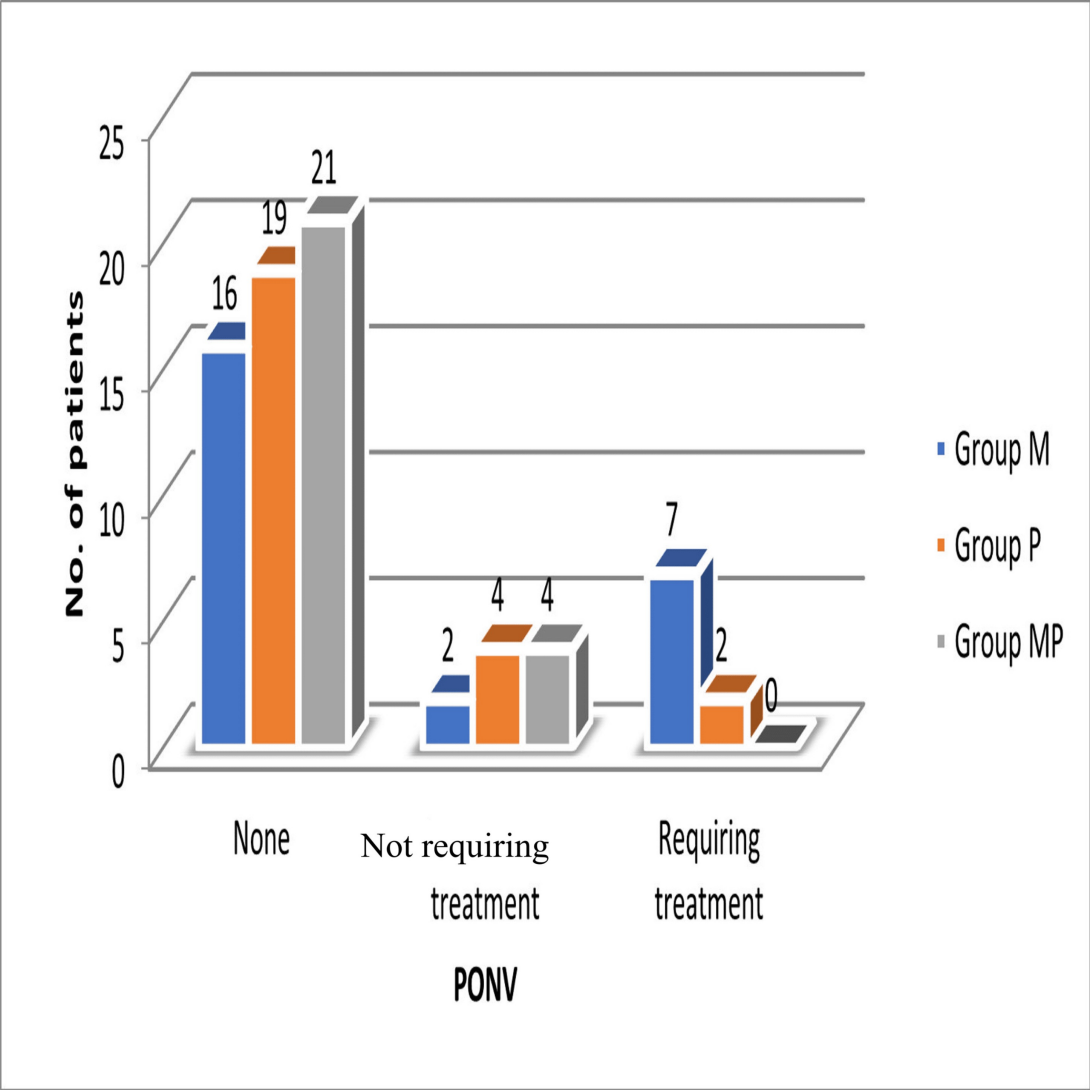
Bar chart depicting the incidence of PONV requiring treatment, highest in Group M and lowest in Group MP. PONV: postoperative nausea and vomiting

No episodes of respiratory depression, oxygen desaturation (<90%), naloxone requirement, significant hypotension, or bradycardia were observed in any group. Intraoperative haemodynamic parameters remained within normal ranges and did not differ significantly between groups. Sedation was assessed using the RSS score. At one hour postoperatively, median RSS values were 3 (interquartile range (IQR) 3-3) in Group M, 4 (IQR 3-4) in Group P, and 4 (IQR 4-4) in Group MP. The Kruskal-Wallis analysis indicated significant group differences (p < 0.001). Pairwise comparisons showed Group MP vs M (p < 0.001) was significant, while Group P vs M did not reach statistical significance due to overlapping distributions despite a higher median in Group P. By two hours, sedation levels had decreased across groups and remained ≤3 through 24 hours. Full distributions and p-values are provided in Table 4. Morphine usage was associated with a higher incidence of postoperative nausea and vomiting requiring treatment in Group M (28%), a lower incidence in Group P (8%), and no cases requiring treatment in Group MP. According to Table 5 and Figure 4, this difference was statistically significant (p = 0.038).

**Table 4:** RSS scores postoperatively. Data are presented as median and IQR (Q1, Q3). Comparison between groups was performed using the Mann-Whitney U test. A p-value of <0.05 was considered statistically significant. RSS: Ramsay Sedation Scale; IQR: interquartile range

Time (hours)	Group M (median, IQR)	Group P (median, IQR)	Group MP (median, IQR)	Overall p (Kruskal-Wallis)	Pairwise comparisons (Mann-Whitney U test with Bonferroni correction)
1	3 (3-3)	4 (3-4)	4 (4-4)	<0.001	MP vs M: p < 0.001; P vs M: not significant; MP vs P: not significant
2	3 (2-3)	3 (2-3)	3 (3-3)	0.212	All not significant
4	3 (2-3)	3 (2-3)	3 (2-3)	0.664	All not significant
8	2 (2-3)	2 (2-3)	2 (2-3)	0.791	All not significant
12	2 (2-2)	2 (2-2)	2 (2-2)	0.930	All not significant
24	2 (2-2)	2 (2-2)	2 (2-2)	1.000	All not significant

**Table 5:** Incidence of PONV requiring treatment. Statistical comparison was performed using the chi-squared test (χ² = 10.14; p = 0.038), indicating a statistically significant difference among groups (p < 0.05). PONV: postoperative nausea and vomiting

Group	Patients requiring treatment for nausea and vomiting (n, %)	P-value
Group M	7 (28%)	0.038
Group P	2 (8%)	
Group MP	0 (0%)	

Patient‑reported outcomes

**Figure 5:**
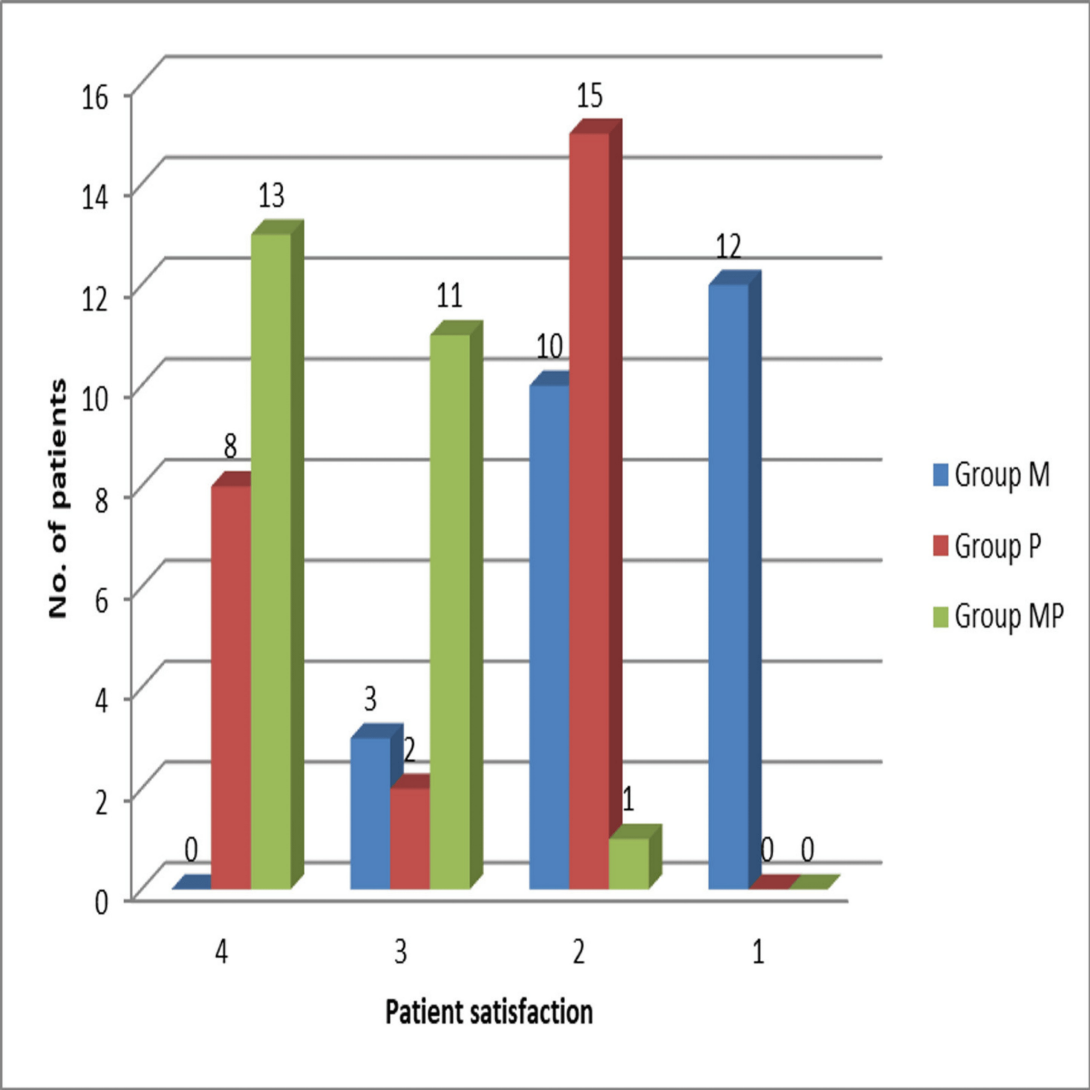
Bar chart showing patient satisfaction scores across groups on a 4-point Likert scale, highlighting greater satisfaction in Group MP.

Patient satisfaction was evaluated using a Likert scale with four points reflecting their perception of pain management. A majority of patients in Group MP (52%) "strongly agreed" that their pain was adequately controlled, compared to 60% of patients in Group P who gave a score of 2 ("disagree") and 48% of patients in Group M who scored 1 ("strongly disagree") (p < 0.001) (Table 6 and Figure 5).

**Table 6:** Patient satisfaction scores (4-point Likert scale). Statistical comparison was performed using the chi-squared (χ²) test, which compares observed and expected frequencies. The test statistic was χ² = 62.14 with 6 df and p < 0.001, indicating a statistically significant difference in satisfaction distribution among the groups. df: degrees of freedom

Satisfaction score	Group M (n, %)	Group P (n, %)	Group MP (n, %)
4 (strongly agree)	0 (0%)	8 (32%)	13 (52%)
3 (agree)	3 (12%)	2 (8%)	11 (44%)
2 (disagree)	10 (40%)	15 (60%)	1 (4%)
1 (strongly disagree)	12 (48%)	0 (0%)	0 (0%)

## Discussion

The present study demonstrated that the preoperative combination of intravenous magnesium sulphate and oral pregabalin significantly reduced both the total dose and the number of bolus doses of morphine required post-thoracotomy. This combination also prolonged the duration of analgesia, decreased the incidence of PONV, and increased patient satisfaction. These effects may be attributed to the blockade of multiple pain pathways: pregabalin acts on the alpha-2-delta subunit of presynaptic calcium channels to reduce calcium influx, thereby decreasing the release of excitatory neurotransmitters such as glutamate, while magnesium sulphate antagonises NMDA receptors, preventing central sensitisation and reducing hypersensitivity to pain.

The primary aim was to reduce opioid consumption and its associated side effects through multimodal analgesia. Our findings align closely with prior work. In thoracotomy patients, Abdelgalil et al. showed that adding pregabalin to magnesium sulphate cut 24-hour morphine consumption to 28.47 ± 5.76 mg versus 33.97 ± 6.34 mg with pregabalin alone, 40.87 ± 4.40 mg with magnesium alone, and 42.20 ± 6.10 mg with placebo; VAS scores were significantly lower at zero and four hours in the pregabalin-containing groups (all patients were on PCA) [7]. Similarly, in arthroscopic rotator cuff repair, Jo et al. reported improved early analgesia and less rescue morphine with the pregabalin-magnesium regimen [11]. In spine surgery, Tavanaei et al. found that the combination group had the lowest 24-hour morphine consumption (7.3 ± 2.9 mg) and lower pain scores than either drug alone or placebo, with less PONV than placebo [12]. Although the absolute difference in 24-hour morphine consumption between groups was modest (3-4 mg), even small opioid reductions are clinically meaningful, as they reduce the risk of opioid-related adverse effects such as PONV, sedation, and delayed recovery. This was reflected in our study, where lower morphine use in the combination group was associated with significantly less PONV and greater patient satisfaction.

Pregabalin by itself has also performed well in thoracic populations. Sattari et al. observed lower VAS scores after thoracotomy, e.g., upon regaining consciousness (mean 4.04 ± 2.31 vs 5.00 ± 1.75; p = 0.01) and at six hours (4.67 ± 2.59 vs 6.04 ± 3.24; p = 0.001), with significantly reduced 24-hour pethidine use [13]. Gaber et al. showed that perioperative pregabalin lowered morphine consumption on day 1 (17.0 ± 4.2 mg vs 20.3 ± 5.1 mg; p = 0.011), day 2 (7.2 ± 2.4 mg vs 9.5 ± 2.6 mg; p = 0.001), and day 3 (3.6 ± 1.6 mg vs 5.1 ± 1.8 mg; p = 0.001), along with fewer adverse effects [14]. These reports reinforce the beneficial pregabalin effects we observed. We acknowledge that the literature reports variable effect sizes for pregabalin across surgical procedures and doses; prior thoracic and non-thoracic studies in our reference list (e.g., Abdelgalil et al. [7], Sattari et al. [13], Gaber et al. [14]) also demonstrate heterogeneity in outcomes. This context helps explain and frame the modest absolute reductions observed in our trial.

Magnesium sulphate alone did not meaningfully reduce morphine consumption in our cohort, likely reflecting the intensity of post-thoracotomy pain. Even so, Ghezel-Ahmadi et al. found a lower Numerical Rating Scale (NRS) pain at rest on postoperative days (POD) 1-8 with perioperative magnesium (e.g., POD 1: 1.28 ± 1.43 vs 2.06 ± 1.48; p = 0.009) and a lower burden of neuropathic pain by the Leeds Assessment of Neuropathic Symptoms and Signs (LANSS) at 30 days (2.1% vs 14.3%; p = 0.031) and 90 days (0% vs 12.2%; p < 0.05) [15].

Importantly, in our study, the pregabalin-magnesium group consistently showed lower VAS pain scores, particularly at one, 3-5, eight, and 12-24 hours postoperatively. While pain was adequately controlled in all groups, most likely due to baseline PCA morphine, the superior pain scores in the combination group highlight its greater efficacy, a finding that parallels the results reported by Abdelgalil et al. [7].

Since both pain severity and opioid consumption are well-recognised contributors to PONV, the reduction in morphine use observed with the pregabalin-magnesium combination likely explains the lower incidence of PONV in this group. In our study, PONV requiring antiemetic treatment occurred in 28% of patients in the magnesium group, 8% in the pregabalin group, and none in the combination group. This pattern is consistent with previous findings by Abdelgalil et al., Jo et al., and Tavanaei et al., all of whom reported reduced PONV rates with this multimodal regimen [7,11,12].

Another notable observation was the increased sedation in the combination group during the early postoperative hours, which is in keeping with the known pharmacological effects of both agents. However, this sedation was not problematic; in fact, patients appeared calmer and less agitated. Statistically significant differences in RSS scores at one and four hours post-surgery reflect this effect, and similar early postoperative sedation with pregabalin-magnesium co-administration has also been described by Abdelgalil et al. [7].

Limitations

The study was limited by its 24-hour follow-up, lack of assessment during coughing or deep breathing, use of a continuous basal PCA infusion, and omission of multimodal analgesics and thoracic epidural comparators. In addition, variable pregabalin doses, systematic respiratory safety monitoring, and serum magnesium levels were not evaluated, which restricts generalisability.

## Conclusions

In patients undergoing thoracotomy, preoperative administration of pregabalin combined with magnesium sulphate significantly reduced the primary outcome of 24-hour total morphine consumption compared to either drug alone. This opioid-sparing effect was accompanied by lower pain scores, less PONV, and greater patient satisfaction without compromising haemodynamic stability. Based on these findings, this combination can be considered as part of a multimodal preoperative analgesic strategy to improve early postoperative pain control in thoracic surgery.
